# Yeast Products Mediated Ruminal Subenvironmental Microbiota, and Abnormal Metabolites and Digestive Enzymes Regulated Rumen Fermentation Function in Sheep

**DOI:** 10.3390/ani12223221

**Published:** 2022-11-21

**Authors:** Manchun Su, Huihui Wang, Huibin Shi, Qiao Li, Yong Zhang, Taotao Li, Youji Ma

**Affiliations:** 1 College of Animal Science and Technology, Gansu Agricultural University, Lanzhou 730070, China; 2 Gansu Key Laboratory of Animal Generational Physiology and Reproductive Regulation, Gansu Agricultural University, Lanzhou 730070, China; 3 School of Agriculture and Forestry Technology, Longnan Teachers College, Chenxian, Longnan 742500, China

**Keywords:** feed supplement, ruminal digestive system, subenvironmental microbiota, small ruminants

## Abstract

**Simple Summary:**

Increasing the proportion of concentrate in the diet is a common strategy to improve the performance of ruminants. However, this may lead to abnormal rumen fermentation, which is a threat to animal health. As a green feed additive, yeast product (YP) is often used to regulate rumen fermentation and maintain animal health, but its mechanism of action on ruminants is unclear. This study was conducted to investigate the effects of YP (yeast culture + yeast live cells) on rumen fermentation parameters, abnormal metabolites and microbiota in sheep. Our findings suggest that YP can improve rumen fermentation parameters, change the activity of rumen digestive enzymes and reduce the concentration of abnormal metabolites. In addition, we found that YP significantly changed the relative abundance of microorganisms attached to rumen feed particles and affected microbial function. This study provides new insights into the regulation mechanism of feed additives on rumen fermentation and improves our understanding of YP on rumen fermentation regulation.

**Abstract:**

Yeast products (YP) are commonly used as rumen regulators, but their mechanisms of action are still unclear. Based on our previous studies, we questioned whether yeast products would have an impact on rumen solid-associated (SA) and liquid-associated (LA) microorganisms and alter rumen fermentation patterns. Thirty 3-month-old male sheep weighing 19.27 ± 0.45 kg were selected and randomized into three groups for 60 days: (1) basal diet group (CON group), (2) basal diet add 20 g YP per day (low YP, LYP group) and (3) basal diet add 40 g YP per day (high YP, HYP group). The results demonstrated that the addition of YP increased rumen cellulase activity, butyrate and total volatile fatty acid (TVFA) concentrations (*p* < 0.05), while it decreased rumen amylase activity and abnormal metabolites, such as lactate, lipopolysaccharides (LPS) and histamine (HIS) (*p* < 0.05). Metagenomic analysis of rumen microorganisms in three groups revealed that YP mainly influenced the microbial profiles of the SA system. YP increased the relative abundance of *R. flavefaciens* and decreased methanogens in the SA system (*p* < 0.05). With the addition of YP, the abundance of only a few lactate-producing bacteria increased in the SA system, including *Streptococcus* and *Lactobacillus* (*p* < 0.05). However, almost all lactate-utilizing bacteria increased in the LA system, including *Megasphaera*, *Selenomonas*, *Fusobacterium* and *Veillonella* (*p* < 0.05). In addition, YP increased the abundance of certain GHs family members, including GH43 and GH98 (*p* < 0.05), but decreased the abundance of some KEGG metabolic pathways involved in starch and sucrose metabolism, biosynthesis of antibiotics and purine metabolism, among others. In conclusion, the addition of YP to high-concentrate diets can change the abundance of major functional microbiota in the rumen, especially in the solid fraction, which in turn affects rumen fermentation patterns and improves rumen digestibility.

## 1. Introduction

The use of high-grain diets to improve animal performance is now a common nutritional strategy adopted by the livestock industry worldwide. However, high levels of concentrates increase the content of rapidly fermentable carbohydrates in the rumen, and the resultant abnormal rumen fermentation decreases pH, leading to a state of subacute rumen acidosis (SARA), which may eventually increase the risk of metabolic disorders and a series of metabolic diseases such as rumen acidosis, hoof laminitis and liver abscesses [1]. Yeast is a probiotic commonly used in ruminant nutrition; it increases feed efficiency by improving rumen fermentation and effectively prevents rumen acidosis. Yeast has also been shown to be effective in restoring microbial balance in the gastrointestinal tract, especially when the animal is in a state of digestive disorder or stress [2,3].

Yeast can improve the productive performance of ruminants and has been reported to increase calf weight, promote calf feed intake and growth rate [4], and significantly increase weight gain in bulls on high-grain diets [5]. However, some studies also report no effect of active dry yeast on growth and feed conversion in sheep [6], and similar results have been obtained in studies on dairy cattle [7]. These inconsistent results indicate that the effect of yeast on animal production performance is inconclusive, and this may be related to the type and dose of yeast products. Research on active dry yeast and yeast cultures in ruminants has been widespread, and some studies report differing effects [8]. However, there have been far fewer studies on the application of the simultaneous addition of live yeast cells and cultures in ruminants. Improvements in the productive performance of ruminants by yeast may be achieved through its influence on rumen microorganisms. Yeast can inhibit the activity of lactic acid-producing bacteria and promote the proliferation of lactic acid-utilizing bacteria, thereby stabilizing the rumen environment by reducing the accumulation of lactic acid [9]. Many studies report that yeast increases the number of *Megasphaera elsdenii*, an important lactic acid-utilizing bacterium, in the rumen of cows fed a high-grain diet [10]. The ability of yeast to promote the growth of fiber-degrading bacteria has been accepted by most scholars, and many studies in dairy and beef cattle show that yeast can increase the abundance of rumen fiber-digesting bacteria [11,12], such as *F. succinogenes*, *R. albus* and *R. flavefaciens*.

Methane abatement is currently a hot topic in ruminant research, and it has recently been shown that yeast can reduce ruminal methane production by stimulating acetate-producing bacteria to consume more hydrogen to produce acetate [13]. It is also reported that yeast reduces intestinal methane production but does not change the number and diversity of methanogens [14]. Most of these studies on the effects of yeast on rumen microbes have focused on dairy and beef cattle, and relatively little research has been performed on sheep. Moreover, most scholars have studied changes in microbial composition as a whole, and few studies have explored the regulatory role of yeast in terms of taxa involved in key metabolic functions, such as lactate metabolism and methane emission.

Our previous studies revealed differences in composition and functional profiles of rumen solid-associated (SA) and liquid-associated (LA) microorganisms, the microbiota involved in fiber degradation are mainly colonized on feed particles, while soluble nutrient-utilizing microorganisms are more prevalent in rumen fluid [15]. Therefore, it is reasonable to assume that yeast uniquely influences the microbiome in the SA and LA system, alters microbial taxa involved in key rumen functions in both systems, and that yeast regulates sheep rumen fermentation through alterations in these key microorganisms. To our knowledge, this may be the first time that metagenomic techniques have been used to study the effects of yeast on ruminants via alterations in rumen SA and LA system microbes. This mode of study allows more precise targeting of microbial taxa with key functions and helps to identify key microorganisms that are less abundant or less variable in conventional studies. We aim to provide new insights into the study of yeast preparations in the regulation of the rumen.

## 2. Materials and Methods

### 2.1. Ethics Approval

All experimental animal procedures were approved by the Animal Care Committee of Gansu Agricultural University (GSAU-AEW-2020-0057).

### 2.2. Animals and Experimental Design

Thirty male Hu sheep lambs with an initial average body weight of 19.27 ± 0.45 kg (3 months old) were selected and randomly divided into three groups (10 sheep/pen; *n* = 10). These lambs were housed in a barn of a medium-scale Hu-sheep breeding farm (Qingyang, Gansu, China) with good ventilation and ad libitum access to clean water (pen: 2.5 × 12 m^2^). All lambs were fed with fresh feed at 0800 and 1700 h daily throughout the 60-day experimental period following a 14-day adaptation period. Lambs were fed daily using a basal diet supplemented with either 0 g (CON group), 20 g (LYP group), or 40 g (HYP group) yeast product (YP) each per day. The yeast products from Phibro Animal Health Corporation, Teaneck, USA, were composed of yeast cultures and live yeast cells (crude protein (CP) ≥ 12.0%, ether extract (EE) ≥ 3.0%, crude fiber (CF) ≤ 5.0%, ash ≤ 3.0%, water ≤ 10.0%, Saccharomyces cerevisiae ≥ 2 × 108 CFU/g). Lambs were fed a total mixed ration (TMR) to meet their nutritional requirements. Dietary ingredients and chemical composition of the basal diet are presented in Supplementary Table S1 (forage: concentrate ≈ 30:70, dry matter (DM) basis). For each group, the YP was accurately weighted and mixed with 1 kg crushed corn and top-dressed to the feed bunk every morning. These powders containing YP are completely licked clean by the lambs before the remaining feed is filled into the feed trough. Finally, three lambs from each group of approximately average weight were slaughtered at a local slaughterhouse (Qingyang, Gansu, China) at 1200 h after receiving their previous afternoon feed on day 61.

### 2.3. Sample Collection and Processing

The rumen contents were filtered through a 4-layer sterile gauze filter, and the pH was measured immediately. Subsequently, three tubes containing 50 mL rumen fluid and 150 g residues were stored at −80 °C until microbial DNA extraction. Another 50 mL of rumen contents were collected and stored in a sterilized container at −20 °C to measure digestive enzyme activity, abnormal metabolites (lactic acid, lipopolysaccharide and histamine) and fermentation parameters. Rumen contents of all lambs were sampled from the left dorsal sac.

Rumen liquid-associated (LA) microorganisms and solid-associated (SA) microorganisms were extracted using the method from a previous publication [15]. After acquiring 50 mL of rumen fluid, it was filtered with four layers of sterilized cheesecloth and centrifuged at 10,000 g for 20 min at 4 °C. The pellet was resuspended in 10 mL phosphate-buffered saline and stored at −80 °C as rumen LA microbiota samples. Precisely 10 g of rumen feed residue was dissolved in a 50 mL centrifuge bottle with 30 mL of normal saline. The plant particles were sedimented by centrifugation for 15 min at 500 g after gentle shaking for 60 s. The supernatant was carefully removed and centrifuged at 10,000 g for 20 min at 4 °C, then transferred sediments to a new 50 mL fresh bottle as the microbial cells (SA1). The remaining residue was resuspended in 50 mL of an anaerobically prepared diluent containing 0.15% (*v*/*v*) Tween-80 (Surfact-Amps, Thermo Scientific™, Shanghai, China), then gently shaken for 60 s and placed on ice for 2.5 h to elute the tightly adherent microbiota. The mixture was centrifuged at 500 g for 15 min then the obtained supernatant was centrifuged again (10,000 g for 20 min at 4 °C) to obtain tightly attached microbial cells (SA2). The SA1 and SA2 microbes were resuspended in 10 mL saline as rumen SA microorganism samples and stored at −80 °C prior to analysis.

### 2.4. Digestive Enzyme Activities and Abnormal Metabolites

The rumen content was placed in an ultrasonic pulp refiner (DH92-IIN, Lawson Scientific Technology Co., Ltd., Ningbo, China) to obtain a 10% homogenization buffer. After centrifugation at 2500 rpm for 10 min at 4 °C, the supernatant was tested for digestive enzymes and abnormal metabolites. Finally, cellulase and amylase enzymes were measured using the colorimetry method according to the manufacturer’s instructions (Biosino Biotechnology Co., Ltd., Beijing, China; Mindray BS-240 automatic biochemical analyzer, Mindray, Shenzhen, China). Concentrations of lactic acid, lipopolysaccharide and histamine were measured using enzyme-linked immunosorbent assays (ELISA) (Biosino Biotechnology Co., Ltd., Beijing, China; microplate reader DR-200BS, Hiwell-Diatek Instruments Co., Ltd., Wuxi, China).

### 2.5. Fermentation Parameters

Determination of ammonia nitrogen by spectrophotometer colorimetric method [16]. Instantly measured rumen pH with a portable pH meter (Model PHB-4, Shanghai Leica Scientific Instrument Co., Ltd., Shanghai, China). Volatile fatty acid (VFA) concentrations were determined as described previously [17]. In brief, 2 mL of thawed rumen content was centrifuged at 5400 rpm at 4 °C for 10 min, and then 0.2 mL of 25% metaphosphoric acid was added to 1 mL of the supernatant for homogenization. The mixed solution was centrifuged at 10,000 rpm for 10 min at 4 °C, and VFAs (acetate, propionate and butyrate) were determined by a gas chromatograph (HP6890N, Agilent Technologies, Wilmington, DE, USA) equipped with an AT-FFAP capillary column (50 m × 0.32 mm × 0.25 µm). The column temperature was held at 60 °C for 1 min, then ramped unrestricted at 5 °C/min to 115 °C, then to 180 °C at 15 °C/min. 

### 2.6. Microbial DNA Extraction and Metagenomic Sequencing

Microbial DNA extraction and metagenomic sequencing methods have been described in detail in our previous studies [15]. Metagenomic DNA was extracted from rumen contents and subjected to concentration determination and integrity assessment, followed by sequencing on the Illumina Hiseq X Ten platform (Illumina, San Diego, CA, USA) from Biomarker Technologies (Beijing, China). Metagenomics sequence data that have been filtered and decontaminated from the host [18] were assembled by Megahit v.1.1.2 [19], and contigs with lengths ≥ 300 bp were selected as the final assembling result. They were used for further gene prediction and annotation. Predicted genes from all samples were gathered together to construct the non-redundant (NR) gene catalog set by setting 95% identity and 90% coverage of the gene with the longer sequences in the clustering. After quality control, clean reads were mapped to the NR gene set [20]. Subsequently, representative sequences of the NR gene set were aligned to the NCBI NR database to obtain annotation results and species abundance. The hmmscan software (v.3.1b2) was used to make the ORFs aligned with the Carbohydrate-active enzymes (CAZy) database with the e-value cutoff of 1e^−5^. Lastly, the Kyoto Encyclopedia of Genes and Genomes (KEGG) pathway annotation approach was performed against the KEGG database using Diamond (v.2018-07-30) with the e-value cutoff of 1e^−5^.

### 2.7. Pyrosequencing Data Accession Number

Raw sample data from Illumina sequencing has been deposited at NCBI. They are available from BioProject. Accession number: PRJNA854684 (Sheep rumen metagenome).

### 2.8. Statistical Analysis

All data were presented as mean with SEM levels. One-way ANOVA was used in the analysis of the rumen fermentation parameters, digestive enzyme activity and abnormal metabolites and was performed using SPSS v.22.0 software (SPSS Inc., Chicago, IL, USA). Differences were considered significant at *p* < 0.05. All necessary analyses were performed using GraphPad Prism v.8.0 software. Metagenomic statistics were performed using the BMKCloud online platform (www.biocloud.net, accessed on 10 August 2021). Taxonomic and functional data were evaluated using the MOTHUR v.1.35.0 program. Principal coordinate analysis (PCoA) results were constructed and visualized by an R package (R ade4 package, v.2.15.3). The significance of the PCoA plot among three groups was tested by permutational multivariate analysis of variance (PERMANOVA). Wilcoxon tests were performed on the differential abundance of the genus, species and CAZymes modules using the stats R package in R software (Version 3.3.1). Linear discriminant analysis (LDA) effect size (LEfSe) analysis was used to determine the biomarkers explaining the differences among CON, LYP and HYP groups. The LDA score cutoff was set at 2.0. Pearson’s correlation coefficient analysis was performed on rumen microbiota, digestive enzyme activity, fermentation parameters and abnormal metabolites. RDA (Redundancy analysis)/CCA (Canonical Correspondence analysis) was used to assess the correlation between rumen microbiota (top genus), treatment groups (CON, LYP and HYP) and environmental factors; results were analyzed and visualized using the R software (Version 2.3).

## 3. Results

### 3.1. Effects of YP on Fermentation Parameters, Abnormal Metabolites and Digestive Enzyme Activity

In order to evaluate the effect of YP on rumen health and digestive function in sheep, rumen fermentation parameters, abnormal metabolites and digestive enzyme activity were measured. As shown in Table 1, there were no significant differences in pH, acetate, propionate and ammonia-N concentration with YP (*p* > 0.05). Ruminal butyrate and TVFA concentrations were greater (*p* < 0.05) in HYP than in CON and LYP. YP had a great effect on rumen digestive enzyme activity, while amylase concentrations were greater (*p* < 0.05) in CON than in LYP and HYP. Cellulase activity in HYP was significantly increased over CON and LYP (*p* < 0.05). Compared with CON, YP significantly decreased lactic acid, LPS and HIS concentration in HYP and LYP groups (*p* < 0.05).

### 3.2. YP Changed the Composition Profiles of Rumen Solid-Associated (SA) and Liquid-Associated (LA) Microbiota

We further investigated the effect of YP on the taxonomy and function of rumen microorganisms. Metagenome sequencing of samples (SA and LA systems) generated 168.14 Gb high quality and no host clean reads, with an average of 9.34 Gb per sample. We assembled 3,022,059 contigs and identified 14,673 species from 2520 genera in the SA system and 2,349,082 contigs and identified 14,472 species from 2633 genera in the LA system (Supplementary Table S2). At the genus level, *Prevotella*, *Bacteroides*, *Ruminococcus*, *Treponema* and *Alistipes* were the most abundant genera of the SA system, while *Prevotella*, *Bacteroides*, *Selenomonas*, *Clostridum* and *Ruminococcus* were the most abundant genera of the LA system (Supplementary Figures S1 and S2). We discovered that YP significantly affected the composition of rumen SA microbes at the genus level (PERMANOVA, Binary-Jaccard distance: R = 0.373, *p* = 0.025; Supplementary Figure S3), but it had little effect on LA microbes (PERMANOVA, Binary-Jaccard distance: R = 0.311, *p* = 0.101; Supplementary Figure S4). To further evaluate the effect of YP on rumen microorganisms, we screened the top 50 genera in SA and LA systems for one-way ANOVA analysis. Results are shown in Table 2; YP had a significant impact on the relative abundance of eight genera in the SA system and five genera in the LA system (*p* < 0.05). Among them, the relative abundance of *Fusobacterium*, *Dialister*, *Ruminococcus*, *Dorea* and *Lactobacillus* in the SA system, and the relative abundance of *Mitsuokella* and *Selenomonas* in the LA system was higher and significantly increased with the addition of YP (*p* < 0.05). However, the relative abundances of *Methanobrevibacter* and *Paraprevotella* in the SA system decreased significantly with the addition of YP.

**Figure 1:**
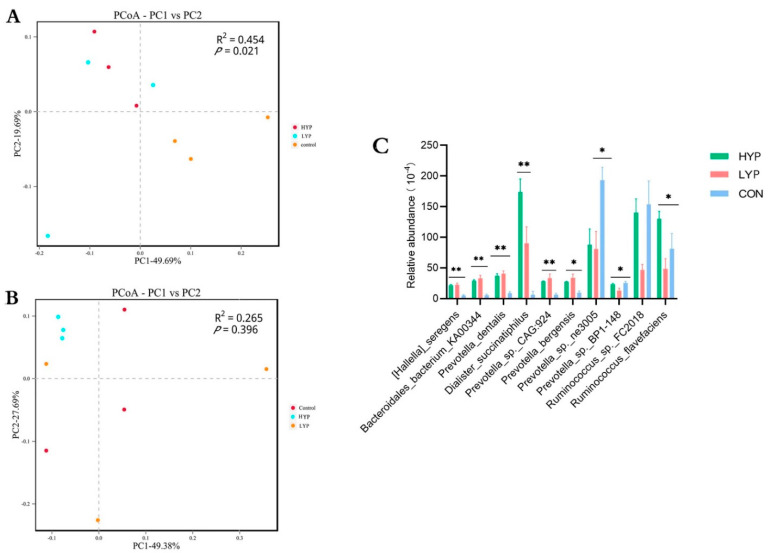
Effects of yeast product on the microbial composition of the rumen. (**A**) PCoA of solid-associated (SA) microorganisms based on Bray–Curtis distance at the species level. (**B**) PCoA of liquid-associated (LA) microorganisms based on Bray–Curtis distance at the species level. (**C**) Effect of YP on species-level microorganisms. * *p* < 0.05 and ** *p* < 0.01.

The beta diversity of microbial communities in CON, LYP and HYP groups was calculated and visualized using Bray-Curtis distance PCoA, PERMANOVA was used to test significance. Results showed that microorganisms of the three groups were distinct in SA systems, but PCoA at the species level illustrated a slight difference in the LA system (Figure 1A,B). Therefore, we focused on the effect of YP on the microbial composition of rumen SA microbiota. At the species level, 14,674 species were identified among the three groups, and a one-way ANOVA was used to compare microbial communities among the three groups. As shown in Figure 1C, the relative abundances of *[Hallella] seregens*, *Bacteroidales bacterium KA00344*, *Prevotella dentalis*, *Dialister succinatiphilus*, *Prevotella sp. CAG:924*, *Prevotella bergensis* and *R. flavefaciens* were considered to have significantly increased after YP supplementation (*p* < 0.05). However, *Prevotella* sp. *ne3005* was significantly decreased with the addition of YP (*p* < 0.05).

**Figure 2:**
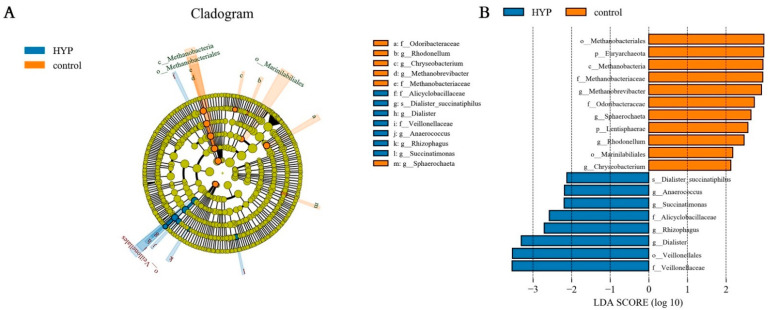
Effects of yeast product on the microbial composition of the rumen (LEfSe analysis). (**A**) Linear discrimination analysis (LDA) effect size (LEfSe) analysis among three groups in the SA system. (**B**) Histogram of the LDA scores computed for microorganisms differentially abundant among three groups of SA microorganisms. LDA scores (log10) > 2.0 are listed.

To further evaluate the differences in microbial communities among the three groups and screen out the specific biomarkers, a linear discriminant analysis (LDA) effect size (LEfSe) was performed. Results showed that there was a significant difference between the HYP and CON groups (Figure 2A,B). Briefly, members of *Veillonellales*, such as *Dialister*, *Succinatimonas* and *Veillonellaceae* were significantly enriched in the HYP group, whereas *Methanobrevibacter* was the characteristic bacterium in the CON group.

### 3.3. YP Changed the Composition of Microorganisms Related to Lactic Acid Metabolism and Methanogens in the Rumen

Lactic acid is an important metabolite of rumen microorganisms, and its high concentration will harm the rumen’s health. To further explore the effect of YP on rumen lactic acid metabolism, we evaluated the changes in microorganisms associated with lactic acid metabolism in SA and LA systems among the three groups. The result is shown in Table 3. The relative abundance of *Streptococcus* and *Lactobacillus* in the SA system increased significantly with the addition of YP (*p* < 0.05); they are usually involved in the production of lactic acid. *Fusobacterium*, an important genus of lactic acid-utilizing bacteria, was significantly increased in the HYP group (*p* < 0.05). In the LA system, the relative abundance of *Mitsuokella* was significantly higher in HYP than in the other two groups (*p* < 0.01). Interestingly, in the LA system, the relative abundance of *Megasphaera*, *Selenomonas*, *Fusobacterium* and *Veillonella*, almost all of the lactic acid-utilizing bacteria, was significantly increased with YP supplementation (*p* < 0.05). 

We further analyzed the effect of YP on methanogens in the rumen. As shown in Table 4, 30 methanogens were identified in the LA system, of which only *Methanothrix* and *Methanobrevibacter* showed a slight difference in the three groups (*p* = 0.064 and *p* = 0.070, respectively). However, in the SA system, six of the 29 identified methanogens decreased significantly with the addition of YP (*p* < 0.05); *Methanobrevibacter* and *Methanosphaera* were the two important methanogens with the highest abundance (Table 4).

### 3.4. YP Changed Functional Profiles of Rumen Solid-Associated (SA) and Liquid-Associated (LA) Microbiota

**Figure 3:**
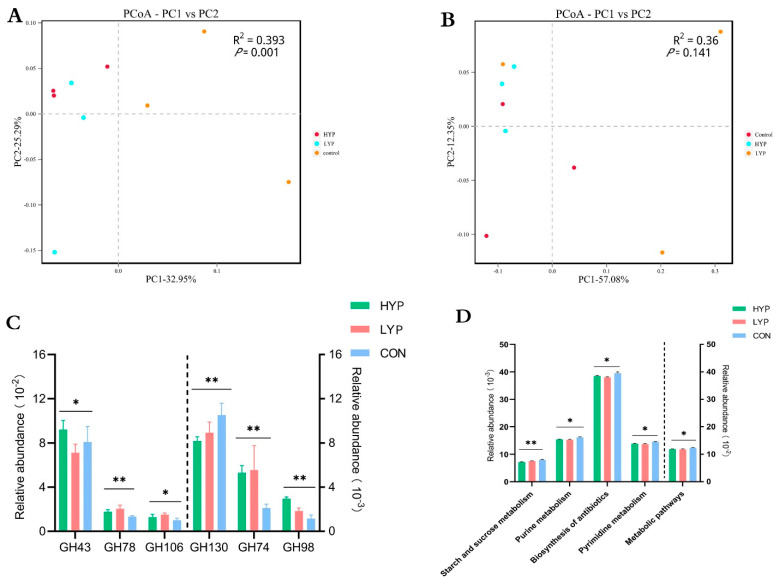
Effects of yeast product on the metabolic function profiles of rumen microorganisms. (**A**) PCoA of solid-associated (SA) microorganisms based on Binary–Jaccard distance in KO level. (**B**) PCoA of liquid-associated (LA) microorganisms based on Binary–Jaccard distance in KO level. (**C**) Comparisons of the gene abundance of the GH family members among three groups in the SA system. (**D**) The functional differences among three groups in SA microorganisms at KEGG-pathway level 3. * *p* < 0.05 and ** *p* < 0.01.

At the gene level, 1,885,514 (SA) and 1,669,804 (LA) non-redundant genesets in the three groups were detected and used to evaluate the effect of YP on functional microbial profiles. As shown in Figure 3A,B, similar to the results of species composition analysis, the PCoA analysis at the KO level showed that YP had limited influence on the microbial function of the LA system (*p* > 0.05), but significantly influenced the SA system (*p* < 0.05). Therefore, we continued to focus on the specific effects of YP on the functional microbial profiles of the SA system. GHs were the most abundant and important members of the CAZymes family, which hydrolyze glycosidic bonds in complex carbohydrates and often assist in the degradation of cellulose, hemicellulose and starch. Here, the relative abundance of GH43, GH78, GH106, GH74 and GH98 were higher in HYP and LYP than in the CON group (*p* < 0.05), and they were usually involved in plant cellulose and lignin degradation. Moreover, these genes corresponded to encode xylanase (EC 3.2.1.8), endoglucanase (EC 3.2.1.4), xyloglucanase (EC 3.2.1.151) and others. On the other hand, the GH130 family, which is involved in mannose metabolism and encodes β-1,4-mannosylglucose phosphorylase (EC 2.4.1.281) and β-1,2-oligomannan phosphorylase (EC 2.4.1.340), was considerably higher in the CON than in HYP and LYP groups (*p* < 0.05, Figure 3C). Furthermore, we evaluated the effect of YP on the KEGG functional potential of SA microorganisms. Metabolic pathways that were significantly different among the three groups at KEGG-level 3 were screened out (*p* < 0.05, Figure 3D). Results showed that five of the top 20 metabolic pathways significantly decreased with YP addition (*p* < 0.05); they are involved in starch and sucrose metabolism, purine metabolism, biosynthesis of antibiotics, pyrimidine metabolism and metabolic pathways.

### 3.5. Correlation Analysis between Rumen Digestive Enzyme, Fermentation Parameters, Abnormal Metabolites and Microbiota

**Figure 4:**
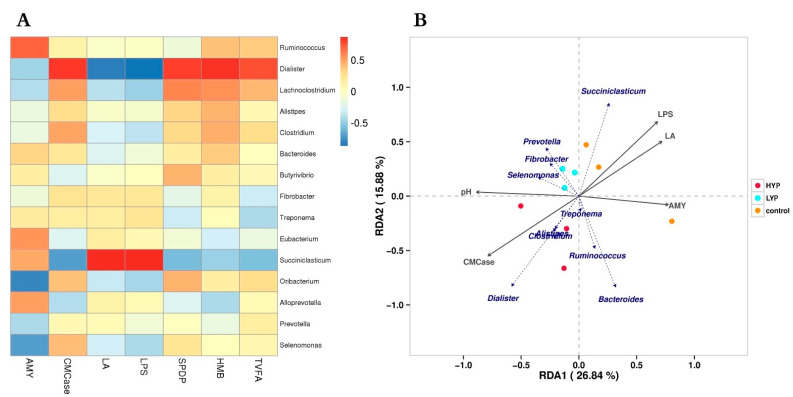
Correlation analyses between the relative abundances of microorganisms and environmental parameters. (**A**) Pearson correlation heatmap differences between digestive enzyme activities, fermentation parameters and microbiota of the solid-associated (SA) system (R > 0.4 or R < −0.4; *p* < 0.05). (**B**) RDA/CCA analysis of the top 10 genera of the SA system and five rumen environmental factors. The relationship between the points of elements in the graph is represented by the distance; the relationship between the rays is represented by the angle, where the obtuse angle represents a negative correlation, and the acute angle represents a positive correlation.

Based on the above results, we continued to pay attention to the relationship between microorganisms in the SA system and other indicators in correlation analysis. The top 15 genera and ruminal digestive enzymes, abnormal metabolites and fermentation parameters were evaluated by regression analysis using Pearson’s correlation coefficient method (R > 0.4 or R < −0.4, *p* < 0.05; Figure 4A). The results showed a correlation between environmental factors and microbiota, but this was not significant (*p* > 0.05). The relative abundance of *Succiniclasticun* was positively correlated with concentrations of lactate and lipopolysaccharide, whereas it was negatively correlated with cellulase. The relative abundance of *Dialister* was positively correlated with the concentration of TVFA, butyrate and cellulase but was negatively correlated with lactate and lipopolysaccharide. The relative abundance of *Oribacterium* and *selenomonas* was negatively correlated with lactate. To further understand the association between microbiota, ruminal digestive enzymes, abnormal metabolites, fermentation parameters and YP, RDA/CCA analysis was used to assess their correlations. Results are shown in Figure 4B; concentrations of lipopolysaccharide, lactic acid and amylase were positively correlated with the CON group and also with the relative abundances of *Succiniclasticum* and *Prevotella*. However, cellulase and pH were positively correlated with the HYP group and also with the relative abundances of *Dialister*, *Clostridium*, *Alistipes*, *selenomonas* and *Treponema*.

## 4. Discussion

Feeding high-grain diets have been used to increase growth rates in ruminants, but it generally increases the incidence of SARA. Appropriate doses of YP may counteract the rumen acidosis of these grain diets. A recent review shows that yeast has a positive effect on rumen fermentation indicators such as pH, VFA and ammonia, and most of our results supported this study [9]. The current study found that the addition of YP significantly increased the concentration of rumen butyrate and TVFA, which is consistent with the results of two studies in dairy cows [21,22]. These changes suggest that YP can improve the rumen fermentation environment and thus improve feed conversion efficiency in sheep. Therefore, we further investigated the effect of YP on abnormal rumen metabolites and digestive enzyme activity. Results showed that YP increased ruminal cellulase activity and decreased amylase activity. The activity of digestive enzymes in the rumen is primarily driven by microorganisms, and the ability of yeast to stimulate the growth of fiber-digesting bacteria is almost universally recognized. This can be attributed to yeast’s ability to provide nutrients that encourage the growth of cellulolytic bacteria, such as *F. succinogenes*, *R. albus* and *R. flavefaciens* [23]. A study on beef cattle also showed that live yeast or yeast cell wall polysaccharides improve the digestibility of ADF and NDF [24], which is similar to our findings. Starch is known to be an important energy source in monogastric animals, but for ruminants, too much starch is a challenge to the rumen. Previous studies in dairy cows have shown that yeast can reduce the relative abundance of amylolytic bacteria, such as *Ruminobacter amylophilus* and *Succinimonas amylolytica*, and thus reduce the digestibility of starch in the rumen, which might partially promote a more neutral rumen pH [25,26]. These results are consistent with the fact that the addition of YP reduced rumen amylase activity. Interestingly, a study on dairy goats found that the relative abundances of GH13-9 and CBM48 (responsible for starch degradation) were reduced in diets with high rumen degradable starch [27]. This leaves us puzzled as to whether yeast affects amylolysis or starch itself causes amylolysis, and the specific mechanisms may require further investigation. Increased concentrations of rumen LPS, HIS and lactate are common during acidosis challenges. Lactic acid accumulation results in a sustained drop in pH [9], while LPS and HIS lead to a systemic inflammatory response characterized by increases in plasma acute-phase proteins, such as serum amyloid-A and haptoglobin [28]. Our findings suggest that yeast can stabilize the rumen environment by reducing the concentration of these abnormal metabolites, with similar results seen in sheep with acidosis [1].

The above results suggest that YP can improve rumen fermentation parameters and maintain rumen health. The results of this study on the growth potential and apparent nutrient digestibility also showed the beneficial effects of yeast in sheep (Supplementary Table S3). The rumen is a fermentation vat containing a large number of microorganisms, which are closely associated with these results. Our previous studies have shown that there are significant differences in composition and function between SA and LA microbes in the rumen [15]. Therefore, we decided to investigate the effect of YP on the microbial composition in these two different systems. At the genus level, *Fusobacterium*, *Dialister*, *Ruminococcus*, *Dorea* and *Lactobacillus* in the SA system and *Mitsuokella* and *Selenomonas* in the LA system were significantly increased following YP supplementation. The mechanism of *Fusobacterium*, a butyrate-producing and lactate-utilizing bacterium, in the rumen is unknown. However, a recent meta-analysis shows that it is significantly enriched in the gut of obese individuals [29], which may imply that *Fusobacterium* plays an important role in weight gain. Distinctively, *Dialister* was almost never present in previous yeast-related studies, but in our study, it was the marker microorganism that differentiated it from the CON group. Several studies on calves have shown that *Dialister* spp. in the rumen or feces is beneficial to the intestinal environment and production performance, such as body weight, feed intake and efficiency [30,31]. These positive effects on ruminants may be owing to the ability of *Dialister* spp. to digest starch and fiber and promote the production of VFAs [32]. *Ruminococcus* is one of the most common genera in the rumen and is known to be an important cellulose-degrading bacterium. Many studies have reported the ability of yeast to increase the relative abundance of *Ruminococcus* [8,33], which is consistent with our findings. *Dorea* is also a genus rarely seen in previous studies, and a study on calves found that *Dorea* is enriched in healthy calves and might be a key microbial marker that can differentiate “healthy” and “unhealthy” (diarrheic) gut microbiota [34]. However, two studies have also reported a higher abundance of *Dorea* in the intestine of obese individuals and Crohn’s patients [29,35]; further studies are needed regarding the effects of this genus on ruminants. *Lactobacillus* is an important probiotic and lactic acid-producing bacteria. Previous studies have shown that yeast can inhibit the growth of lactic acid-producing bacteria, such as *Lactobacillus* [9], which is different from our results. This may be owing to the high-concentrate diet and the fact that the rumen may still be developing in three-month-old lambs, which is supported by a study related to weaned calves [36]. Notably, *Lactobacillus* is important for the gastrointestinal health of young hosts by preventing the growth of pathogenic microorganisms [37]. In addition, *Lactobacillus* may provide substrates for lactic acid-utilizing bacteria to promote the growth of such bacteria, which is supported by our data, as shown in Table 3. In the SA system, we observed a significant decrease in the abundance of *Methanobrevibacter* and *Paraprevotella* with the addition of YP. Some studies report that yeast can reduce the abundance of *Methanobrevibacter* [38], suggesting a potential role for yeast in methane production. There are few studies addressing *Paraprevotella* in ruminants: one study in dairy cows shows a negative correlation between *Paraprevotellaceae* and feed efficiency [39], while another study shows that *Paraprevotella spp.* were enriched in the guts of patients with Behcet’s disease [40], all of which may demonstrate a potential negative effect of *Paraprevotella* on ruminants. Although YP had little effect on the microbiota of the rumen LA system, *Mitsuokella* and *Selenomonas* also increased significantly with the addition of YP. Interestingly, *Mitsuokella* is an important lactic acid-producing bacterium while *Selenomonas* is an important lactic acid-utilizing bacterium, and they are closely related to rumen lactic acid accumulation; the balance between them will be analyzed in depth in later sections.

Similar to the genus-level results, at the species level, YP had a significant effect on microbes in the SA system, so we focused on species changes in SA. The abundance of *[Hallella] seregens*, *Prevotella dentalis* and *bergensis* increased in the rumens of beef cattle supplemented with monensin and eventually led to an increase in propionate concentration [41]. Similar results may suggest that yeast can promote the shift of rumen fermentation to the propionate type through changes in SA species, ultimately resulting in beneficial effects on the host. *Bacteroidales* are considered the core rumen microbiota, and their genomes encode a broad range of plant polysaccharide degradation capabilities, thus being able to degrade cellulose [42]. Notably, among the populations increased by the addition of YP, the most abundant species were *Dialister succinatiphilus* and *R. flavefaciens*. As mentioned above, *Dialister* spp. can use starch and fiber to produce VFAs, thereby improving the production performance of ruminants. *R. flavefaciens* is a very important cellulose-degrading bacterium in the rumen, and many studies have reported the growth-promoting effect of yeast on this bacterium [1,3,26]. *Prevotella* sp. *ne3005* decreased significantly with the addition of YP at the species level, and we found no relevant reports of this rumen bacterium in other studies. *Prevotella ruminicola* is known to be an important starch- and protein-degrading bacterium, and it has been reported that yeast can reduce the abundance of rumen starch-utilizing bacteria and maintain rumen pH at a healthy level [26], which is consistent with our results. However, it is also reported that live yeast can increase the relative abundance of amylolytic microorganisms [8]; therefore, the specific mechanism may need further study. Members of *Veillonellales* are unique microorganisms enriched in the HYP group, *Dialister* and *Megasphaera* are the main genera of *Veillonellaceae*, and we have discussed the beneficial role of *Dialister* in animal production above. *Megasphaera* acts as an important lactic acid-utilizing bacterium, and it has been shown that yeast can encourage its growth to influence ruminal lactate accumulation [9]. *Methanobrevibacter* is the most abundant genus of methane producers in the rumen, which has been reported to be negatively associated with animal performance [39,43].

The current results showed that changes in microbial communities were related to lactate metabolism and methane production, and lactate accumulation and methane emissions are also key points in ruminant studies, so we further investigated the effect of YP on these bacteria. Yeast can stabilize rumen pH via inhibiting activities of lactate-producing microorganisms, such as *Streptococcus bovis* and *Lactobacillus*, and promoting the proliferation of lactate-utilizing bacteria, thereby reducing lactic acid accumulation in the rumen [9,44]. However, our results showed that the abundance of *Streptococcus* and *Lactobacillus* in SA, and *Mitsuokella* in LA, increased significantly with the addition of YP. Interestingly, the increased abundance of these lactate-producing bacteria did not result in increased ruminal lactic acid concentrations. We speculated that YP might disrupt the balance between lactic acid production and consumption, which is manifested by an increase in almost all lactate-utilizing bacteria in the LA system. Methane is a powerful greenhouse gas that can be produced by methanogenic bacteria in the rumen through a series of redox reactions [45]. Methane not only increases the global greenhouse effect but can also cause animals to lose more than 10% of their energy [3]. Recent studies have shown that *Methanobrevibacter* is enriched in the rumens of low-efficiency cows, and the methanogens pathway is also upregulated in low-efficiency cows [46]. These results demonstrate the negative impact of methanogenic bacteria on ruminants. Many studies have shown that yeast can alter rumen fermentation, reduce the abundance of methanogens and ultimately decrease methane emissions [47,48]. However, it is also reported that active yeast has no significant effect on the abundance of methanogenic archaea [7]. Notably, yeast has been shown to reduce methane emissions but does not affect the abundance of methanogenic bacteria in the rumen fluid [3,14]. Interestingly, our results may explain this phenomenon. We found that only the abundance of methanogens in the SA system changed, which may be because methanogens use H_2_ produced by cellulose-degrading bacteria to produce methane [49]. Our previous studies demonstrated that cellulose-degrading bacteria mainly colonize feed pellets [15], which leads to a directed aggregation of methanogens on solid particles in the rumen. Inhibition of methanogens by yeast may lead to excessive accumulation of H_2_, and *Selenomonas* spp. has been shown to consume H_2_ by shifting fermentation to propionate [50]. *Selenomonas* may be a key microorganism in the role of yeast’s regulation of high-concentrate diets in the rumen, both as a propionate producer using H_2_ to improve energy utilization and as an important lactate-utilizing bacterium. Its important role in dairy cattle feed efficiency has been demonstrated by Xue et al. [46].

Our study showed that YP changed the rumen microbial composition, especially in the solid fraction and that changes in microbiota may lead to changes in the profiles of metabolic function. GHs hydrolyze glycosidic bonds in complex carbohydrates and often assist in the degradation of cellulose, hemicellulose and starch [15]. GH43_17 abundance is usually involved in plant xylan degradation [17]. GH98 is an important component of the cellulosome complex, a structure that degrades cellulose and lignin, and it is mainly involved in encoding xylanase [51]. The abundance of these GH family members increased with the addition of YP in the HYP group, which may suggest that YP promotes the degradation of cellulose and hemicellulose. These results are consistent with an increased abundance of fiber-degrading bacteria, such as *R. flavefaciens*, in the HYP group. In particular, our results on apparent nutrient digestibility in sheep showed that YP was able to enhance the digestibility of ADF and NDF (Supplementary Table S3), possibly owing to the altered cellulose-degrading microorganisms and the increased abundance of GH family members. GH130 is a member of the oligosaccharide-degrading enzymes family [52], encoding mainly some enzymes in mannose metabolisms, such as β-1,4-mannosylglucose phosphorylase (EC 2.4.1.281) and β-1,2-oligomannan phosphorylase (EC 2.4.1.340). The decreased abundance of GH130 with YP addition may imply inhibition of oligosaccharide metabolic activity in the rumen. Furthermore, KEGG functional taxonomy showed that starch and sucrose metabolism, purine metabolism, biosynthesis of antibiotics and others were enriched in the CON group. We hypothesize that the rapid degradation of nutrients, such as starch in high-concentrate diets, alters the rumen environment, leading to the death of gram-negative bacteria, which releases harmful substances such as LPS and histamine [1]. Sheep combat harmful microbes by boosting antibiotic synthesis and adapt to this change by boosting purine and pyrimidine metabolism to restore new microbial structures, while yeast can moderate these dramatic fluctuations and keep the rumen healthy. Notably, the use of metagenomics to investigate the relationship between microbial and host performance parameters may not be the ideal option. Numerous studies have shown that metatranscriptomics may be better suited to uncover the associations between rumen microbial function and host performance [46,53]. Therefore, the effect of yeast on the relationship between rumen microbial metabolic function and sheep production performance needs to be further studied.

**Figure 5:**
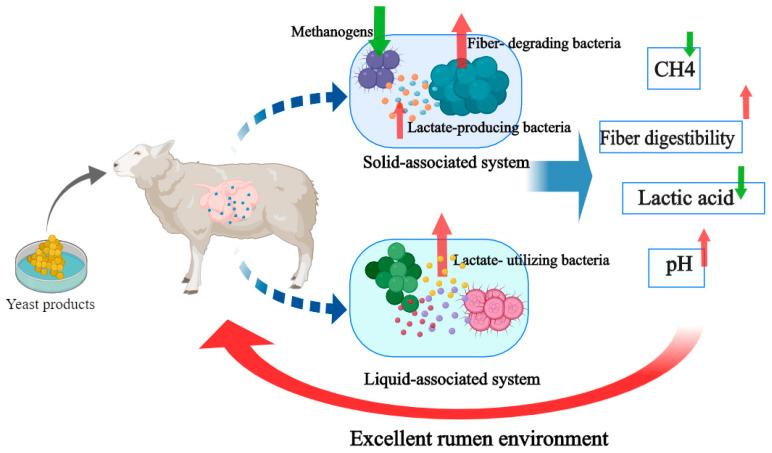
Yeast product regulates rumen fermentation by changing rumen microorganisms.

Our results suggest a mechanism of YP regulation of the rumen in sheep under high-concentrate diets, as shown in Figure 5. Yeast alters the rumen microbiota by providing nutrients or through oxygen consumption. In the SA system, YP reduced the abundance of methanogens and increased the abundance of fiber-degrading bacteria and some lactate-producing bacteria. In the LA system, yeast mainly increased the abundance of lactate-utilizing bacteria. These changes in microorganisms may lead to reduced methane emission, reduced lactate accumulation and increased fiber digestibility, thus stabilizing the rumen environment and improving energy utilization in sheep

## 5. Conclusions

In summary, in the SA system, YP increased the relative abundance of *R. flavefaciens* and several genera of lactate-producing bacteria and decreased the abundance of six identified methanogens. In the LA system, YP increased the abundance of important lactate-utilizing bacteria, including *Megasphaera*, *Selenomonas* and *Fusobacterium*. These alterations in the microbiota may have affected rumen fermentation and ultimately increased YP concentrations of butyrate and TVFA, improved the activity of rumen digestive enzymes and decreased the concentrations of rumen abnormal metabolites, such as lactic acid, LPS and HIS. Based on the results, we conclude that the addition of 40 g/d YP may be more beneficial to sheep rumen health under high-concentrate feeding mode. This study provided new insights into microbiota-mediated regulation of rumen fermentation by feed additives and provided a more systematic theoretical explanation for the role of yeast in high-concentrate diets for ruminants.

## Figures and Tables

**Table 1:** Effects of yeast product on fermentation parameters, abnormal metabolites and digestive enzyme activity.

Item	CON	LYP	HYP	SEM	*p*-Value
pH	6.35	6.45	6.49	0.04	0.182
Acetate, mmol/L	50.11	49.16	57.16	3.04	0.086
Propionate, mmol/L	19.53	22.04	23.00	1.55	0.065
Butytate, mmol/L	15.66 ^b^	16.32 b	19.65 ^a^	0.73	<0.01
TVFA, mmol/L	85.30 ^b^	87.51 b	99.81 ^a^	3.20	0.020
Amylase, U/g.prot	427.77 ^a^	379.87 b	394.87 ^b^	5.60	<0.01
Cellulase, U/ml	23.74 ^b^	26.07 ^b^	29.21 ^a^	0.83	0.011
Ammonia, mg/dL	12.55	11.19	11.10	0.89	0.565
lactic acid, mmol/L	5.46 ^a^	3.57 ^b^	3.12 ^b^	0.03	<0.01
LPS, KEU/ml	43.85 ^a^	32.29 ^b^	21.73 ^c^	2.10	<0.01
HIS, ng/ml	6.17 ^a^	3.71 ^b^	2.20 ^c^	0.05	<0.01

^a,b,c^ Means within a row followed by different lowercase letters differ significantly from each other (*p* < 0.05). CON: lambs that received a basal diet; LYP: lambs that received a basal diet supplemented with 20 g/d yeast products; HYP: lambs that received a basal diet supplemented with 40 g/d yeast products; TVFAs: total volatile fatty acids; LPS: Lipopolysaccharide; and HIS: Histamine.

**Table 2:** Effects of yeast product on the top 50 species of relative abundance at the genus level in sheep rumens.

Item	CON	LYP	HYP	SEM	*p*-Value
Solid-associated Microorganism					
*Methanobrevibacter*	44.78 ^a^	9.07 ^b^	11.95 ^b^	2.93	<0.01
*Fusobacterium*	3.56 ^b^	5.40 ^b^	9.06 ^a^	0.46	<0.01
*Dialister*	8.43 ^b^	111.52 ^a^	216.24 ^a^	20.17	<0.01
*Ruminococcus*	389.02 ^a^	151.53 ^b^	406.60 ^a^	44.61	0.013
*Streptococcus*	4.78 ^b^	4.66 ^b^	5.61 ^a^	0.02	0.024
*Dorea*	3.59 ^b^	5.49 ^ab^	8.69 ^a^	0.87	0.038
*Lactobacillus*	6.33 ^b^	10.30 ^a^	9.18 ^a^	0.80	0.040
*Paraprevotella*	54.28 ^a^	16.65 ^b^	17.01 ^b^	1.95	0.047
Liquid-associated Microorganism					
*Mitsuokella*	17.65 ^b^	5.72 ^c^	42.14 ^a^	1.19	<0.01
*Selenomonas*	110.45 ^b^	73.18 ^b^	276.76 ^a^	24.81	0.024
*Escherichia*	14.57 ^b^	40.83 ^a^	10.61 ^b^	3.38	0.026
*Megasphaera*	12.18 ^ab^	5.28 ^b^	19.56 ^a^	2.83	0.046
*Fusobacterium*	5.32 ^a^	2.85 ^b^	6.93 ^a^	0.82	0.048

^a,b,c^ Means within a row followed by different lowercase letters differ significantly from each other (*p* < 0.05). CON: lambs that received a basal diet; LYP: lambs that received a basal diet supplemented with 20 g/d yeast products; HYP: lambs that received a basal diet supplemented with 40 g/d yeast products. The relative abundance was normalized to 10,000 for the sample.

**Table 3:** Effects of yeast product on the relative abundance of microorganisms related to lactic acid metabolism.

Genus ID	Description	CON	LYP	HYP	SEM	*p*-Values
Solid-associated microorganism						
*Streptococcus*	Lactic acid-producing bacteria	4.78 ^b^	4.66 ^b^	5.61 ^a^	0.16	0.024
*Butyrivibrio*		53.80	55.77	54.92	8.92	0.989
*Lactobacillus*		6.33 ^b^	10.30 ^a^	9.18 ^a^	0.80	0.040
*Mitsuokella*		13.47	31.96	19.14	7.60	0.310
						
*Megasphaera*	Lactic acid-utilizing bacteria	8.58	20.29	22.37	3.25	0.051
*Selenomonas*		55.47	204.30	102.50	41.17	0.127
*Fusobacterium*		3.56 ^b^	5.40 ^b^	9.06 ^a^	0.46	<0.01
*Veillonella*		1.85	2.70	5.28	0.71	0.084
*Propionibacterium*		0.07	0.05	0.05	0.02	0.883
Liquid-associated microorganism						
*Streptococcus*	Lactic acid-producing bacteria	4.60	3.96	4.99	0.26	0.077
*Butyrivibrio*		35.37	27.42	34.04	5.96	0.666
*Lactobacillus*		7.52	6.65	9.11	1.59	0.599
*Mitsuokella*		17.65 ^b^	5.72 ^b^	42.14 ^a^	4.27	<0.01
						
*Megasphaera*	Lactic acid-utilizing bacteria	12.18 ^ab^	5.28 ^b^	19.56 ^a^	2.83	0.046
*Selenomonas*		110.45 ^b^	73.18 ^b^	276.76 ^a^	38.32	0.024
*Fusobacterium*		5.32 ^b^	2.85 ^b^	6.93 ^a^	0.82	0.048
*Veillonella*		2.10 ^b^	1.08 ^b^	3.37 ^a^	0.40	0.041
*Propionibacterium*		0.14	0.12	0.03	0.06	0.598

^a,b^ Means within a row followed by different lowercase letters differ significantly from each other (*p* < 0.05). CON: lambs that received a basal diet; LYP: lambs that received a basal diet supplemented with 20 g/d yeast products; HYP: lambs that received a basal diet supplemented with 40 g/d yeast products. The relative abundance was normalized to 10,000 for the sample.

**Table 4:** Effect of yeast product on methanogens in sheep rumens.

Genus ID	CON	LYP	HYP	SEM	*p*-Value
Solid-associated microorganism					
Methanobrevibacter	44.78	9.07	11.95	2.93	<0.01
Methanohalophilus	0.02	0.02	0.08	0.01	<0.01
Methanosphaera	1.15	0.37	0.52	0.08	<0.01
Methanomethylovorans	0.02	0	0	0.00	<0.01
Methanoculleus	0.1	0.04	0.02	0.01	<0.01
Methanoplanus	0.08	0	0.01	0.01	0.015
Methanothrix	0.1	0.05	0.02	0.01	0.080
Liquid-associated microorganism					
Methanothrix	0.02	0.07	0.01	0.01	0.064
Methanobrevibacter	9.65	35.14	12.65	4.65	0.070

CON: lambs that received a basal diet; LYP: lambs that received a basal diet supplemented with 20 g/d yeast products; HYP: lambs that received a basal diet supplemented with 40 g/d yeast products. The relative abundance was normalized to 10,000 for the sample.

## Data Availability

The data supporting the reported results and conclusions can be found in the submitted figures and tables. The Illumina sequencing raw data for our samples have been deposited into the NCBI. The raw data are available under BioProject, accession number: PRJNA854684 (Sheep rumen metagenome). Additional research materials and protocols that are relevant to the study are available from the corresponding author upon reasonable request.

## Supplementary Materials

The following supporting information can be downloaded at: https://www.mdpi.com/article/10.3390/ani12223221/s1, Table S1: the ingredients and nutrient composition of the basal diet (DM basis); Table S2: summary statistics of sample metagenomic data; Table S3: effect of yeast products on the apparent digestibility of nutrients in sheep; Figure S1: distribution of solid-associated microbial composition at the genus level in the three groups; Figure S2: distribution of liquid-associated microbial composition at the genus level in the three groups; Figure S3: the PERMANOVA analysis of solid-associated microorganisms among the three groups at the genus level; Figure S4: the PERMANOVA analysis of liquid-associated microorganisms among the three groups at the genus level.
