# Supplementary figures and images for: Yeast Products Mediated Ruminal Subenvironmental Microbiota, and Abnormal Metabolites and Digestive Enzymes Regulated Rumen Fermentation Function in Sheep
Source: Animals (Basel). 2022 Nov 21;12(22):3221. doi: 10.3390/ani12223221 (PMC9686794; doi:10.3390/ani12223221)

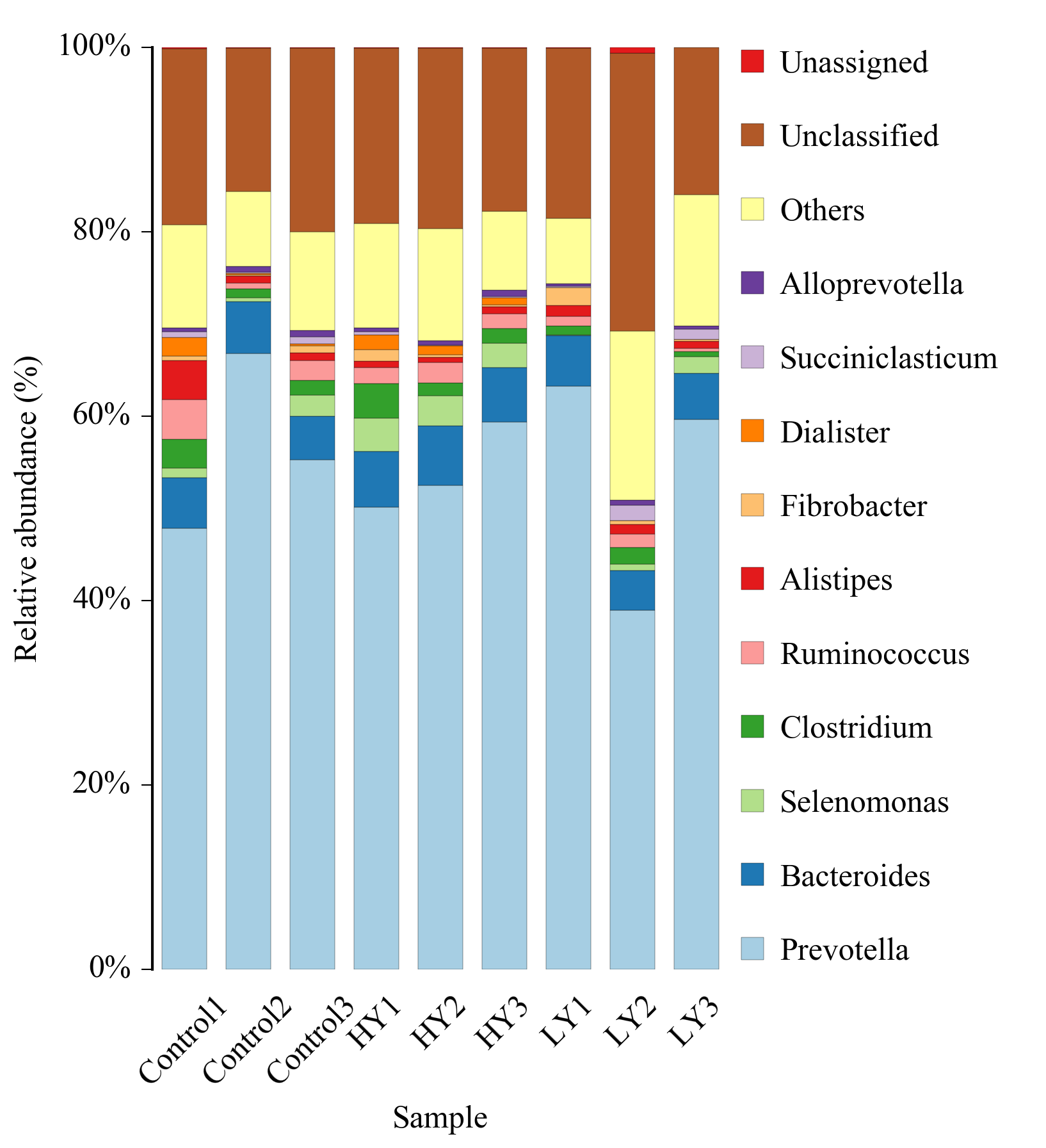

Supplement: Supplementary file 1 [file animals-12-03221-s001.zip › supplementary figure S2.png]

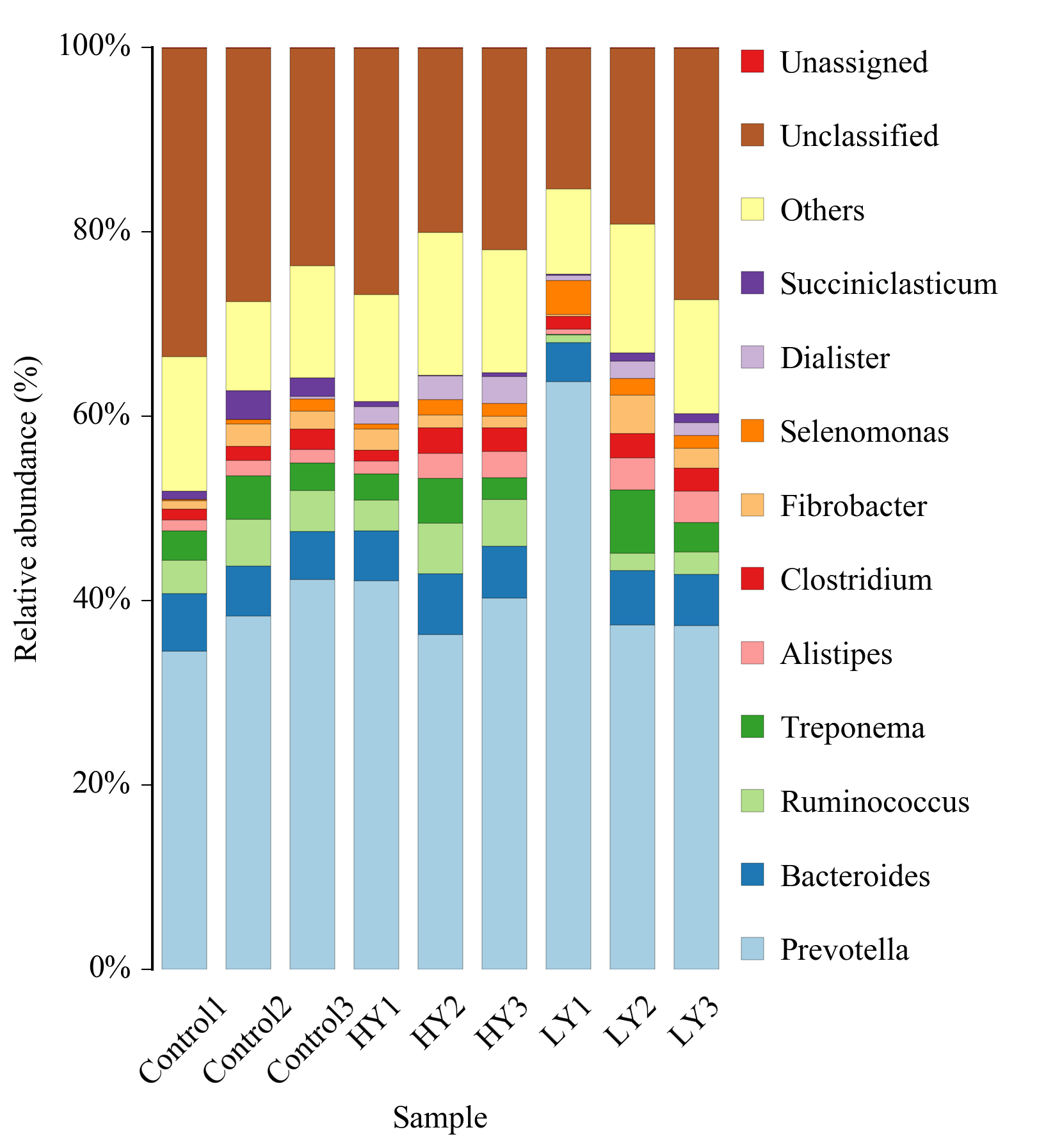

Supplement: Supplementary file 1 [file animals-12-03221-s001.zip › supplementary figure S1.png]

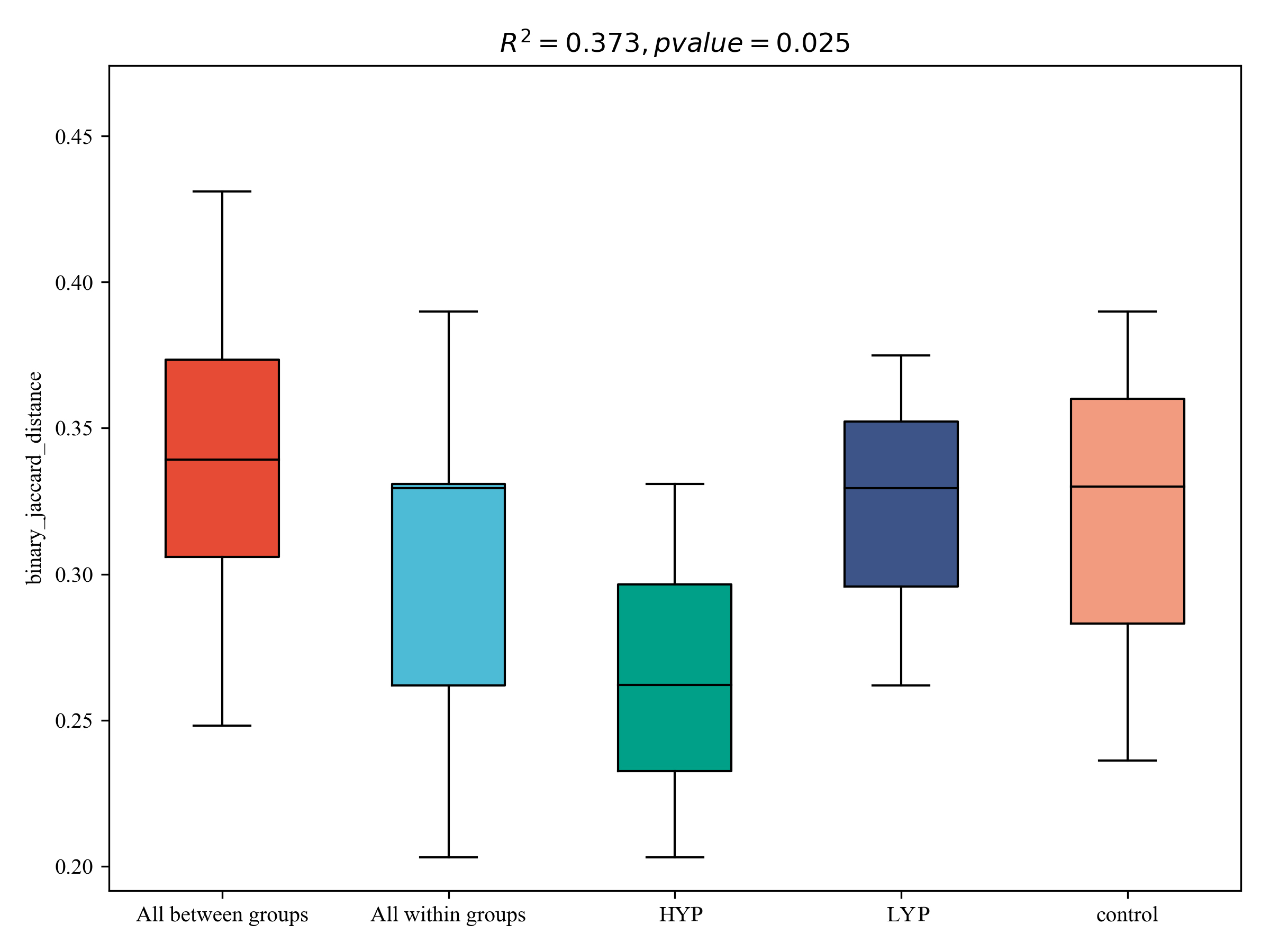

Supplement: Supplementary file 1 [file animals-12-03221-s001.zip › supplementary figure S3.jpg]

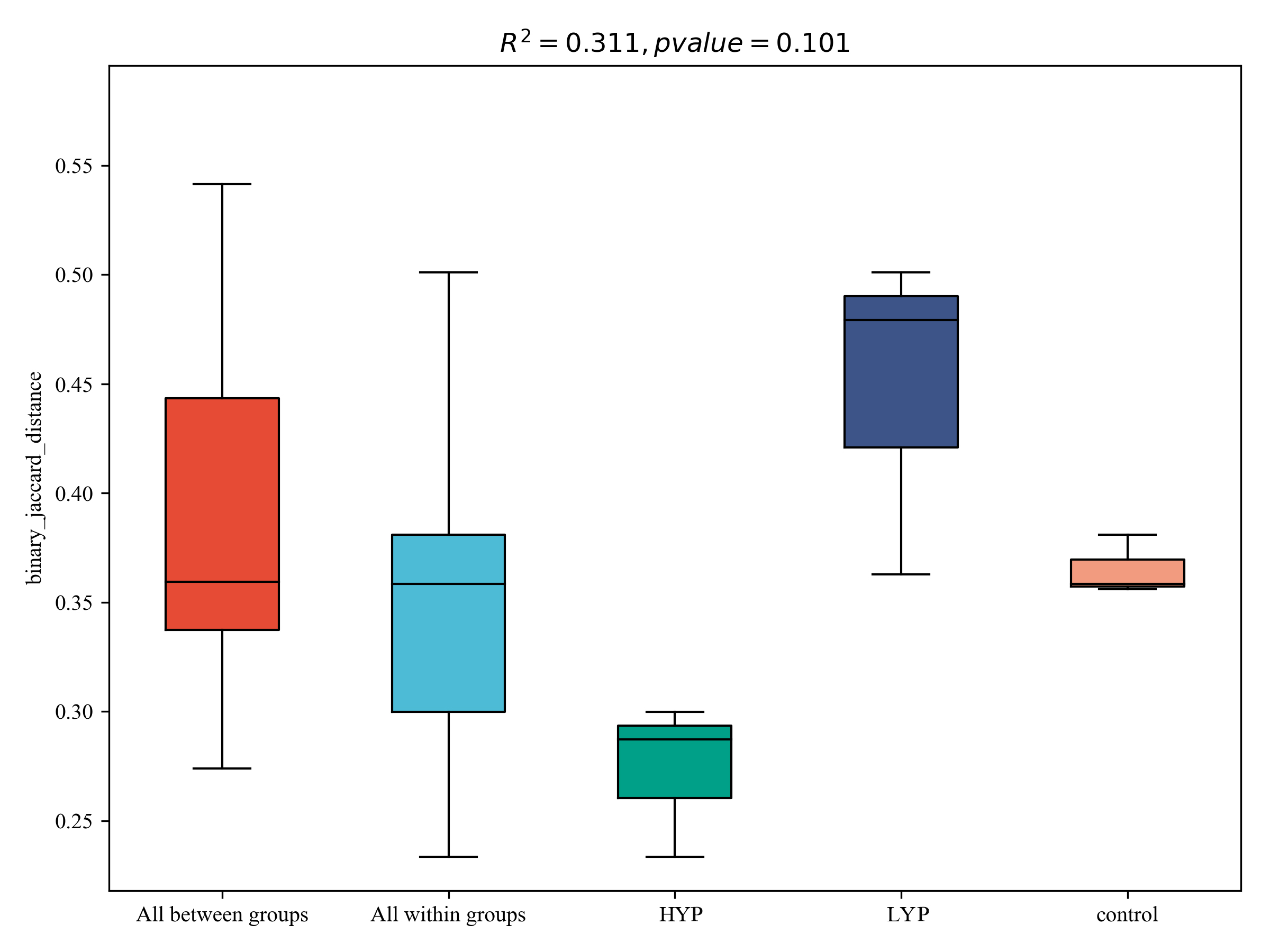

Supplement: Supplementary file 1 [file animals-12-03221-s001.zip › supplementary figure S4.jpg]
